# A magnetic nanoparticle-based microfluidic device fabricated using a 3D-printed mould for separation of *Escherichia coli* from blood

**DOI:** 10.1007/s00604-023-05924-7

**Published:** 2023-08-18

**Authors:** Agnieszka Jóskowiak, Catarina L. Nogueira, Susana P. Costa, Alexandra P. Cunha, Paulo P. Freitas, Carla M. Carvalho

**Affiliations:** 1 Centre of Biological Engineering, University of Minho, Campus de Gualtar, 4710-057 Braga, Portugal; 2 LABBELS –Associate Laboratory, Braga and Guimarães, Portugal; 3 International Iberian Nanotechnology Laboratory, Av. Mestre José Veiga S/N, 4715-330 Braga, Portugal; 4 Instituto de Engenharia de Sistemas e Computadores – Microsistemas e Nanotecnologias (INESC MN) and IN – Institute of Nanoscience and Nanotechnolnology, Rua Alves Redol, 9, 1000-029 Lisbon, Portugal

**Keywords:** Microfluidic, 3D-printed, Magnetic nanoparticles, *Escherichia coli*, Bacteriophage receptor binding protein (RBP), Blood

## Abstract

Herein, A microfluidic device is described, produced with a 3D-printed master mould that rapidly separates and concentrates *Escherichia coli* directly from whole blood samples, enabling a reduction in the turnaround time of bloodstream infections (BSIs) diagnosis. Moreover, it promotes the cleansing of the blood samples whose complexity frequently hampers bacterial detection. The device comprises a serpentine mixing channel with two inlets, one for blood samples (spiked with bacteria) and the other for magnetic nanoparticles (MNPs) functionalized with a (bacterio)phage receptor-binding protein (RBP) with high specificity for *E*. *coli*. After the magnetic labelling of bacteria throughout the serpentine, the microchannel ends with a trapping reservoir where bacteria-MNPs conjugates are concentrated using a permanent magnet. The optimized sample preparation device successfully recovered *E*. *coli* (on average, 66%) from tenfold diluted blood spiked within a wide range of bacterial load (10^2^ CFU to 10^7^ CFU mL^−1^). The non-specific trapping, tested with *Staphylococcus aureus*, was at a negligible level of 12%. The assay was performed in 30 min directly from diluted blood thus presenting an advantage over the conventional enrichment in blood cultures (BCs). The device is simple and cheap to fabricate and can be tailored for multiple bacterial separation from complex clinical samples by using RBPs targeting different species. Moreover, the possibility to integrate a biosensing element to detect bacteria on-site can provide a reliable, fast, and cost-effective point-of-care device.

**Graphical abstract:**

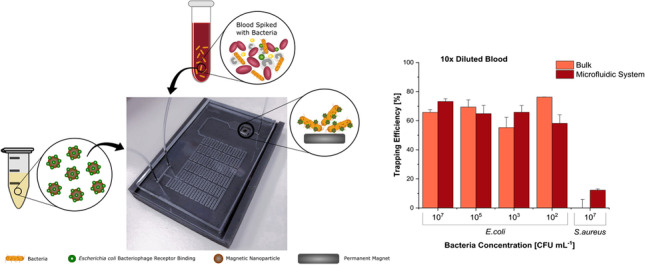

**Supplementary Information:**

The online version contains supplementary material available at 10.1007/s00604-023-05924-7.

## Introduction

Bloodstream infections (BSIs) are among the major causes of morbidity and mortality in hospitals worldwide. They are mainly caused by Gram-positive and Gram-negative bacteria and less frequently by fungi. These infections often lead to the development of sepsis—an organ dysfunction due to a dysregulated inflammatory response of the patient’s organism to pathogens present in blood [1, 2]. Although new molecular methods, i.e. polymerase chain reaction (PCR), fluorescence in situ hybridization (FISH), or matrix-assisted laser desorption ionization time-of-flight mass spectrometry (MALDI-TOF–MS) are being introduced to speed up the diagnostic process, the traditional blood cultures (BC) followed by plate sub-culturing remain the gold standard [2]. Since the concentration of bacteria that can cause sepsis is at very low levels, most of the diagnostic techniques described in the literature do not use blood directly collected from patients but require a prior step of BC [3]. In this case, the collected blood is loaded in standard bottles with rich media to increase the bacterial concentration to detectable levels. The time for BC positivity is on average 16 h but the standard incubation time is 5 days before being discarded as negative [3, 4]. Moreover, BCs can only detect viable and cultivable organisms, and thus are prone to false negatives, especially when the sample is taken from a patient that has already been treated with antibiotics or if the invading pathogen is of slow growth [5, 6]. This imposes a serious threat as with each passing hour of the infection, the survival rate drops by almost 8% [7].

Microfluidic lab-on-a-chip (LOC) solutions are miniaturized systems that help to integrate more than one function on a single platform. These devices are gaining their way into the medical world since they offer several advantages as compared to standard laboratory procedures, such as low cost per assay, small sample and reagents volumes, and a high surface-to-volume ratio that significantly improves efficiency and often reduces time when compared to bulk assays [8]. They are also an important step in the development of point-of-care (POC) devices—self-contained, low-power consumption devices that can be used by medical personnel at the patient’s bedside, providing fast, accurate, and thorough information on patient’s health [9].

Although microfluidic platforms present several advantages, their production can entail high costs namely in the fabrication of the master mould, which is further employed to replicate the device in a cheap, biocompatible polymer, with polydimethylsiloxane (PDMS) being the most commonly used [10]. Conventional fabrication of the master mould using photolithography implies access to cleanroom facilities and is labour-intense, time-consuming, represents significant costs, and limits the height of the fabricated channels [10]. Recently, 3D-printing has emerged as a viable alternative to overcome these hurdles providing ease of fabrication and time reduction, especially for structures with larger dimensions, different heights or complex 3D topography [10]. Most of the 3D-printing techniques employed for master mould fabrication use UV-curable resins as higher print resolutions can be achieved guaranteeing mould surface smoothness and better definition of the printed structures with micron scale features [10]. An example is stereolithography (SLA), where a 3D design can be produced by exposing the resin to a laser beam in a specific sequence, building up the design layer-by-layer in a single process. The resolution of the printed structure depends on the resin composition and the size of the exposing laser beam [11].

Bacterial separation/concentration from whole blood using LOC devices can potentially eliminate the current need for BC enabling a reduction in the turnaround time of BSI diagnosis and contributing dramatically to ameliorating patient outcomes. Nevertheless, the development of such platforms for BSIs faces big challenges due to low loads of pathogens and the complexity of blood samples with components having similar physical features to bacteria [12]. One of the methods to concentrate target molecules from complex matrices in LOC devices that have gained significant interest over recent years is the application of magnetic nanoparticles (MNPs). Nevertheless, the development of this type of system for bacterial concentration in blood is rather limited, with most of the described devices being complex or not performing all the steps of bacterial separation on-chip. Also, most of these devices were tested with high concentrations of bacteria that are not clinically relevant for BSIs [12]. Moreover, these systems are mainly focused on trapping wide spectra of microorganisms from blood [13, 14], and thus need a time-consuming extra step to properly identify the bacteria. The devices that provide specific bacterial capture normally use MNPs functionalized with antibodies [15–17], which are expensive and unstable probes that frequently present cross-reactivity [18].

Receptor-binding proteins (RBPs) from (bacterio)phages are an emerging alternative to standard recognition molecules that can be used coupled with MNPs for bacterial concentration. These proteins are responsible for the recognition and binding of the virus to specific receptors present on the bacterial surface. As the phages normally infect only one species of host bacteria, the RBPs present high binding specificity and offer low production cost, facility for genetic modification, and high chemical and physical resistance [19]. Although the effective use of these proteins as affinity molecules has been reported for different pathogens [19, 20], to the best of our knowledge, their application in microfluidic platforms for human specimen analysis has only been described by our group [21]. Overall, the requirement still exists for a cost-effective, fast, selective, and reliable platform to timely respond to the needs of targeting BSIs, especially, in intensive care units (ICUs).

In this paper, we describe a microfluidic sample preparation device, fabricated using a 3D-printed mould that efficiently uses MNPs to label and trap *Escherichia coli* cells in blood samples. *E*. *coli* was chosen as the model bacterium as it is one of the key pathogens responsible for BSIs in adults and the most common cause of early-onset sepsis in neonates [22]. The device is composed of a serpentine mixing channel with two inlets for introducing the blood sample (spiked with the pathogen) and the MNPs functionalized with a phage RBP (Gp17) which has been recently described as very specific for *E*. *coli* [20]. The channel ends with a trapping chamber where specifically labelled bacterial cells are separated from the whole blood and concentrated using an external magnet. The MNP-bacteria conjugates are then washed and eluted. The total time for bacterial separation from blood is around 30 min. This fully on-chip sample preparation step represents a significant improvement, timewise, when compared to the gold standard BC.

## Materials and methods

### Device fabrication

All microfluidic channels were designed using AutoCAD 2019 (Autodesk, USA) and exported as printer-compatible STL files.

The master moulds for microfluidic structures were fabricated by SLA. The designs were printed on FormLabs Form 2 printer, on the highest resolution (25 µm), using FormLabs Grey Resin photopolymer. Thereafter, following the manufacturer’s protocol, the printed elements were washed in an isopropanol bath for 10 min and cured in the UV oven at 60 °C for 1 h.

Due to issues with the polymerization of the final elastomer channel structure in direct proximity to the mould, the prints were further subjected to an additional heating step at 65 °C. The optimal time was addressed by testing several heating periods, namely 12 h, 24 h, 48 h, and 72 h. After cooling, the mould was used for channel fabrication without any further treatment.

To assess the effect of the channel geometry on the mixing efficiency, several test serpentines were fabricated (Fig. S1A). The final channel design resulting from the optimization of the test structures to best suit the assay needs is represented in Fig. S1B. The drawer-type device holder with a fixed 5 mm × 5 mm × 5 mm neodymium permanent magnet (supermagnete), located underneath the trapping chamber, was also fabricated using the SLA.

The microfluidic structures were fabricated with Sylgard-184 silicone elastomer kit (Dow Inc.) at a 12:1 ratio of polydimethylsiloxane (PDMS) to the curing agent. Filled moulds were placed in the oven at 65 °C for 1.5 h to cure. Ready PDMS structures and corresponding glass slides surfaces were activated using oxygen plasma (plasma cleaner PDC-002-CE) and sealed immediately. To ensure proper bonding, the sealed structures were placed in the oven at 65 °C for 1 h and only used after at least 12 h.

### Evaluation of the printed structures

The dimensions of the printed test structures were measured using a contact profilometer (KLS Tencor P-16), taking three measurements at different points of each of the structures. The mean and standard deviation were calculated. The mixing efficiency in sealed serpentine PDMS structures was tested using yellow and blue commercial food colorants, diluted 1:4 with Milli-Q water for better discernibility of the results.

### Simulations of the mixing efficiency in the microfluidic structures

Mixing efficiency in microfluidic structures was simulated with COMSOL Multiphysics as time-dependent study using Laminar Flow (*spf*) and Transport of Diluted Species (*tds*) physics.

### Bacterial growth conditions and blood samples

The clinical isolates *E*. *coli* HB106 and *Staphylococcus aureus* HB12 provided by the Hospital of Braga (Portugal) were used as the target and negative control bacteria, respectively. The strains were routinely grown overnight in tryptic soy broth (TSB) (Liofilchem) at 37 °C under agitation (120 rpm) or tryptic soy agar (TSA) plates, obtained by adding 12 g/L of agar (Liofilchem) to TSB. The optical density (OD) of the bacterial cultures (1 mL) were adjusted to 0.5, centrifuged (9000 rpm, 10 min) and the pellet resuspended in 1 mL of 0.1 M phosphate buffer (PB) (pH 7.2). For blood testing, the bacterial pellet was resuspended in 1 mL of tenfold diluted (in 0.1 M PB) anonymized remnant blood specimens (EDTA-anticoagulated). Additionally, as a reference blood sample, the bacterial pellet was also resuspended in tenfold diluted (in 0.1 M PB) certified lyophilized human whole blood (BRC-635, Sigma-Aldrich). Serial dilutions to the desired bacterial concentrations were performed (10^2^ to 10^7^ CFU mL^−1^) in the same solution that were previously resuspended.

### Magnetic trapping assays

#### Preparation of the functionalized MNPs

Fifty microliter of commercial 250 nm Ni–NTA-coated MNPs (Nanomag‐D, Micromod, 4.9 × 10^11^ particles mL^−1^) were washed twice with 500 µL of 0.1 M PB Tween 20 (Scharlab) (0.05%, v v^−1^) by removing the supernatant after trapping the particles with a magnetic concentrator (Dynal‐Biotech). Then, 500 µL of the recombinant RBP Gp17, expressed and purified as described by Costa et al. [20], at a final concentration of 5 µM was added to the MNPs and incubated in an orbital shaker at 500 rpm, 20 °C for 2 h. After MNP functionalization, the above-mentioned washing procedure was performed to remove the supernatant containing the unbound protein. In the next step, 500 µL of a 5% (w v^−1^) bovine serum albumin (BSA) (Sigma-Aldrich) solution in 0.1 M PB was added to the MNPs to block the remaining free surface and incubated at the same conditions as during functionalization for 1 h. The supernatant was discarded and the MNP-RBPs conjugates were washed with 0.1 M PB Tween 20 (Scharlab) (0.05% v v^−1^), resuspended in 50 µL 0.1 M PB, and kept at 4 °C as stock. For comparison purposes, 50 µL of commercial 250 nm streptavidin-coated MNPs (Nanomag‐D, Micromod, 4.9 × 10^11^ MNPS mL^−1^) were functionalized with 500 µL of a biotinylated *E*. *coli* polyclonal antibody (LS-C56164, LSBio) at a final concentration of 50 μg mL^−1^, following the same procedure described above.

#### Magnetic separation of bacteria in bulk

 For bulk experiments, magnetic trapping was performed by incubating 200 µL of* E*. *coli* cells (10^2^ to 10^7^ CFU mL^−1^) or *S*. *aureus* cells (10^7^ CFU mL^−1^) prepared as previously described (in PB or tenfold diluted blood) with 2 µL of MNP-RBP for 1 h at 20 °C, 500 rpm in an orbital shaker. Supernatants corresponding to the unbound fraction (UF) were collected and the MNPs were washed twice with 200 µL of 0.1 M PB Tween 20 (Scharlab) (0.05%, v v^−1^), following the same procedure described above for the functionalization of MNPs. The supernatants were collected for evaluation of the wash fractions (WF). All fractions were plated on TSA for direct enumeration of the bacterial loads and the bacterial trapping efficiency (TE) (%) was calculated using the following formula: $\mathrm{TE}= (\mathrm{IF}-(\mathrm{UF}+\mathrm{WF}))/\mathrm{IF }\times 100$; where (in CFUs) the IF (initial fraction) are the bacteria initially added, UF (unbound fraction) is the supernatant corresponding to the bacteria that were not captured during the assay, and WF (wash fraction) are the bacteria collected during the washing steps. The same procedure was followed for the assays where the MNPs functionalized with the antibody or the RBP were compared regarding their TE for *E. coli* or *S. aureus* cells (10^6^ CFU mL^−1^).

#### Magnetic separation of bacteria in the microfluidic device

 For the experiments in the microfluidic serpentine channel, 100 µL of *E*. *coli* suspension with pre-defined concentrations (10^2^ to 10^7^ CFU mL^−1^) or *S*. *aureus* (10^7^ CFU mL^−1^) in 0.1 M PB was introduced through the larger inlet at a flow rate of 20 µL min^−1^, controlled by a syringe pump (NE-1010, New Era Pump Systems, Inc.). Simultaneously, 10 µL of functionalized MNPs (MNP-RBP), diluted to a final volume of 50 µL with 0.1 M PB, was introduced through the smaller inlet at a flow rate of 10 µL min^−1^. After both solutions have entered the serpentine, 0.1 M PB buffer was passed at identical flow rates for the washing steps. The bacterial magnetic trapping was achieved by fixing the permanent magnet below the reservoir, close to the microfluidic structure outlet. For the separation of bacteria from blood samples, a similar procedure was followed with the only difference being that bacterial suspensions were resuspended in whole blood samples diluted tenfold with 0.1 M PB (preparation described in the above section “Bacterial growth conditions and blood samples”). The samples corresponding to the fraction IF (bacteria loaded in the device) and fractions UF and WF (collected from the magnetic chamber, before and after the washing step, respectively) were plated for CFUs quantification, and the trapping efficiency TE was calculated as described previously for the bulk experiments.

Both blood samples initially loaded into the serpentine and collected from the magnetic chamber were diluted and stained with Trypan blue solution (Sigma-Aldrich) and the blood cells (BCs) were counted using a Neubauer chamber. The purity of the captured sample (MNPs with bacteria) in terms of BCs was assessed using the formula: (1-[BCs] _collected_/[BCs] _initial_) × 100.

### Statistical analysis

All data are represented as mean ± SD (standard deviation). For Fig. 4, multiple comparisons of means were performed using two-way ANOVA followed by Tukey’s multiple comparisons test (*p*-value < 0.001).

## Results and discussion

### Master mould

To rapidly optimize the serpentine design and reduce the speed and production costs of the master mould preparation, a 3D-printing technique, namely the SLA method, was used. The dimensions of the channels of the printed moulds showed to be in good agreement with the designed dimensions for both 100 µm and 200 µm high channels (Fig. S2). When using the 3D-printed structure as a master mould, it was observed that the polymerization efficiency of the PDMS is affected by the proximity to the surface of the printed structure. Poor or complete lack of polymerization in direct contact with the mould is a common problem associated with 3D-printed structures. Two mechanisms have been suggested as possible explanations: sequestration of the polymerization catalyst by some resin components [23] or release of its inhibitors from the printed elements [24]. A recent study by Venzac et al. on 16 different SLA resins described in detail the underlying mechanism and presented the most common approaches to tackle this polymerization issue such as UV-post curing, temperature treatment, use of solvents, silanization, or another type of coating [25]. Since high temperatures, as well as additional UV exposure time, can deform large structures and affect mould durability, and to avoid additional labour-intense coating steps in the laboratory, we have used a simple, lower temperature-based approach, which proved to be sufficient to eliminate this problem. More specifically, the mould was exposed to a temperature of 65 °C, identical to that of PDMS curing, for various time periods. The best results were achieved after leaving the mould in the oven for 72 h. No further surface treatment was necessary. The casted PDMS after a 1.5 h curing time at 65 °C was fully polymerized and detached easily from the master mould. The optimized methodology allows to use the oven for PDMS curing, does not induce material stress to the moulds and avoids surface treatment that in some cases may hinder the biocompatibility of the resulting PDMS structure.

### Serpentine mixer

**Fig. 1:**
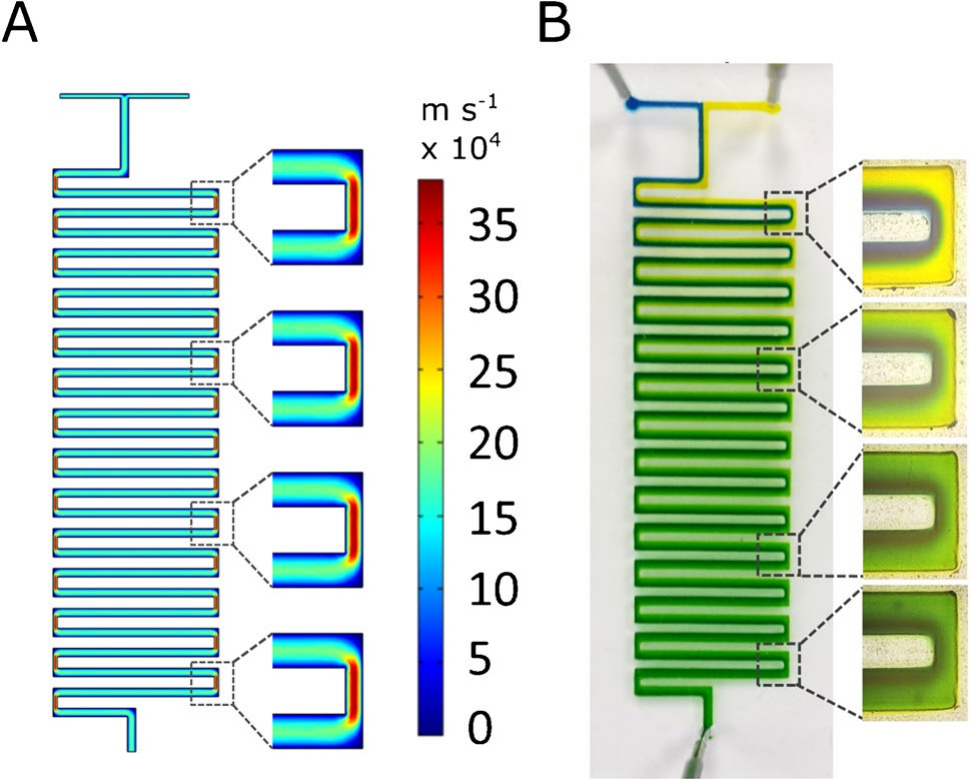
**A** COMSOL simulation results for the flow velocity profile mixing. **B** Tests with food colorants in the microfluidic structures with 600 µm channel width, 200 µm channel height, and 300 µm curve width

The optimized design of the serpentine microfluidic channel (Fig. S1B) was a result of the combination of COMSOL simulations and hands-on experience with different serpentine layouts (Fig. S1A). As the fluid transport in microchannels follows the laminar flow regime, the mixing occurs almost solely by molecular diffusion. A common method to induce a more efficient chaotic mixing is the introduction of obstacles inside the structure [26]. For the development of our device, this approach was excluded to avoid possible MNPs aggregation on these internal structures. Instead, a geometry-based mixing enhancement was selected, namely a square wave geometry as it is described as having the most significant impact on the mixing efficiency [27]. For promoting chaotic liquid movements and disrupting the laminar flow, modifications in the channel height and serpentine curves’ width were introduced. Additional structures with a higher number of curves were also fabricated. The devices were subjected to COMSOL simulations and further tested with food colorants to evaluate the influence of the geometry on the mixing efficiency. The results from these optimizations are described in detail in the “Supplementary information” section (Figs. S3, S4, S5, S6). Overall, the tests with the food colorants corroborated the simulations, showing that the mixing was more efficient in the higher structures and that the reduced curve width was the condition that most contributed to improving the mixing. As depicted in Fig. 1A, this geometry creates a nozzle effect i.e. when entering the reduced dimension area the fluid is comprised and accelerated, and expands and decelerates when entering again the wider area [28]. This enforces chaotic liquid movement, hence mixing, as can be observed in Fig. 1B with the food colorants.

**Fig. 2:**
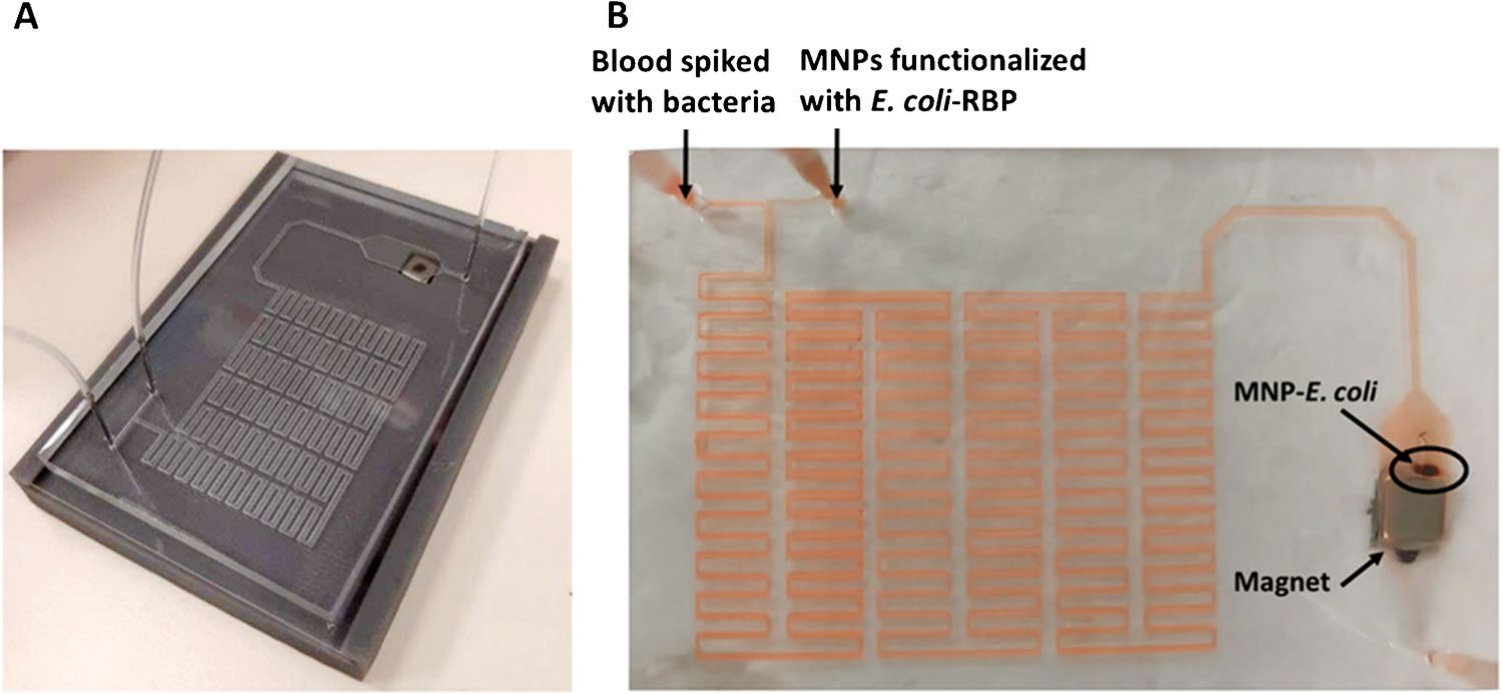
**A** Microfluidic device fixed in the dedicated support holding a permanent magnet. **B** Micromixer during the assay with blood spiked with bacteria introduced at the 450-µm-wide inlet and MNP-RBP introduced at the 225-µm inlet. The MNP-bacteria conjugates were trapped by a permanent magnet placed under the trapping reservoir

Also, the length of the serpentine channel was optimized by further increasing the number of curves (Fig. S1B) both for promoting the mixing and the retention time of the fluids inside the channel to guarantee sufficient reaction time for the binding of bacteria to the functionalized MNPs. The optimized channel (Fig. 2) was further used for bacterial trapping assays.

### Bacteria separation in bulk and in the microfluidic system

**Fig. 3:**
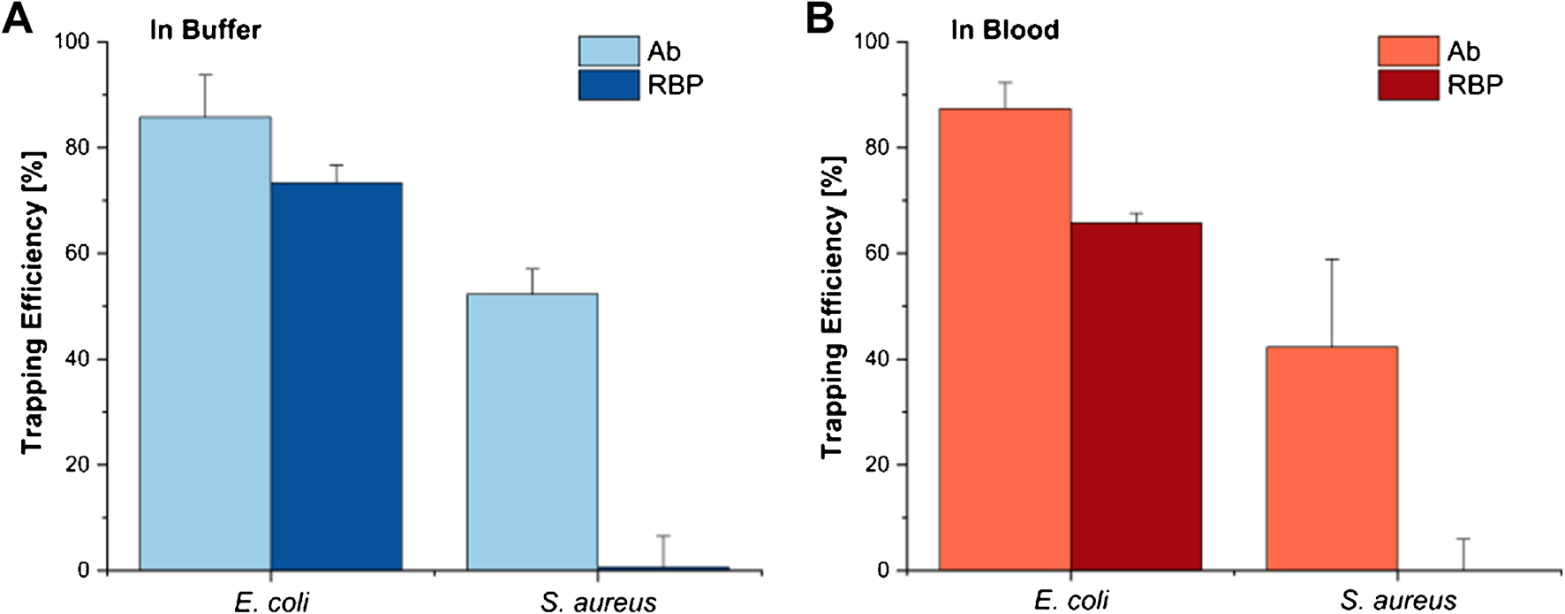
Bacterial trapping efficiency (TE) in buffer (**A**) and in tenfold diluted blood (**B**) for MNPs functionalized with a polyclonal antibody and MNPs functionalized with the RBP. For both the target (*E*. *coli*) and negative control (*S*. *aureus*), a cell concentration of 10^6^ CFU mL^−1^ was used. All the assays were performed in bulk

Preliminary experiments were conducted to assess the potential of the recombinant phage RBP Gp17 [20] in comparison with a polyclonal antibody for the specific magnetic capture of *E*. *coli* in buffer and diluted blood (Fig. 3). Although both molecules showed high trapping efficiencies for *E*. *coli* (more than 65%), the RBP-functionalized MNPs showed non-specific binding to *S*. *aureus* (negative control, non-target bacteria) in blood contrarily to the antibody that presented around 42% non-specific binding, thus offering a cost-effective and more specific alternative. For these reasons, the RBP Gp17 was chosen for further assays to guarantee proper bacteria trapping and identification. We have previously demonstrated the high specificity of RBPs for other nosocomial pathogens such as *Enterococcus*, *S*. *aureus*, *Pseudomonas* *aeruginosa*, and *Klebsiella* *pneumoniae* [21, 29, 30]. Moreover, Gp17 has been already tested in different human specimens enabling the specific detection of *E*. *coli*, thus providing a powerful probe for the diagnosis of different types of infections caused by *E*. *coli* [20].

The bacteria trapping efficiency using MNPs functionalized with Gp17 was tested in the micromixing/trapping serpentine and compared with the corresponding experiments in bulk. For both types of assays, *E*. *coli* (10^2^ to 10^7^ CFU mL^−1^) and *S*. *aureus* (as negative control) suspensions in PB or in tenfold diluted whole blood were used.

**Fig. 4:**
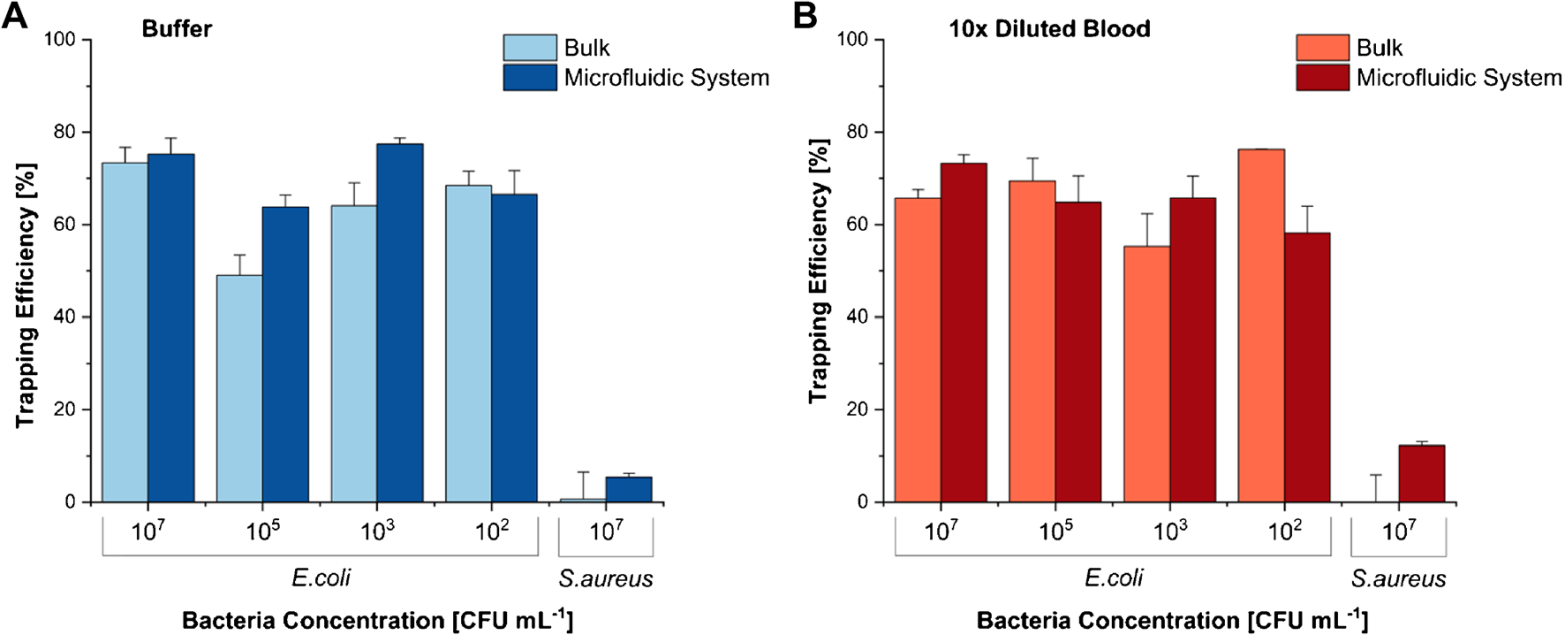
Comparison of bacteria trapping efficiency (TE) in bulk and in the microfluidic structure for assays performed with spiked buffer samples (**A**) and spiked tenfold diluted blood samples (**B**) for target bacteria, *E*. *coli*, at different concentrations and the negative control, *S*. *aureus*, at 10^7^ CFU mL^−1^. Pairwise comparisons between the TE of the *E*. *coli* samples (from 10^2^ to 10^7^ CFU mL^−1^) and the negative control (*S*. *aureus*) show statistically significant differences (*p* < 0.001)

Figure 4A shows that the *E*. *coli* cells at all concentrations were efficiently captured and isolated from buffer in both bulk and serpentine experiments (on average 64% and 71%, respectively). The results obtained in the experiments performed in diluted blood (Fig. 4B) were similar to the buffer assays, being the capture of *E*. *coli* on average 67% in bulk and 65% in the serpentine. This shows that the overall capture of the target pathogen was not affected by the complexity of the blood samples [12]. For the serpentine experiments, the coefficient of variation inter-assays was 7%, which indicates that it is highly reproducible. Moreover, the same analysis performed in commercial reference blood indicates that the bacterial capture efficiencies are comparable with the ones obtained for the spiked human blood specimens, which confirms the consistency of the experiments performed with this type of samples (Fig. S7). The assays in bulk and serpentine with the non-target pathogen (*S*. *aureus*) revealed just a neglected bacterial capture (less than 12%) with values significantly different (*p* < 0.001) from the ones obtained for all the *E*. *coli* concentrations tested (10^2^ to 10^7^ CFU mL^−1^). Moreover, in a real-case scenario, this unspecific binding is not problematic since the MNPs-bacteria conjugates collected from the serpentine have to be further analysed by diagnostic methodologies (e.g. conventional culturing, molecular-based methods, biosensors) for pathogen identification [2].

The results presented herein show the feasibility of using the developed serpentine microfluidic system to replace the traditional out-chip procedures for blood sample preparation prior BSIs diagnosis. These are laborious, time-consuming, and/or require specialized equipment and technicians and cannot be used as POC. The use of MNPs in this process can present several advantages such as being simple, fast, not expensive, and providing the concentration and separation of the target from complex samples.

There are very few MNPs-based microfluidic platforms described in the literature that only use external permanent magnets to separate bacteria from blood [13–17, 31]. In comparison with these systems, our device presents several advantages (Table 1) such as simplicity, low cost, and easy fabrication due to the use of a 3D-printed mould, high specificity for *E*. *coli* provided by the RBP, ability to recover low-bacterial concentration from blood and rapidness. Moreover, the MNPs-conjugates collected from the magnetic chamber presented a 98% depletion of BCs in relation to the blood sample initially loaded in the device. This is of utmost importance since the complexity of blood frequently hampers the efficiency of several methodologies currently used for the detection of pathogens. Examples include the interference of blood components in molecular methods and the autofluorescence of erythrocytes that affect optical-based diagnostic techniques [12, 21].

**Table 1:** Comparative analysis of our device with other microfluidic systems that use permanent magnets for *E*. *coli* separation from blood

Reference	3D printing (yes/no)	Target bacteria	Recognition molecule	Specific detection	Specificity testing	Sample	Assay on-chip	Assay time	LOD (CFU/mL)
Our device	Yes (mould)	*E*. *coli*	RBP	Yes	*S*.* aureus*	Diluted Human blood	Mixing, reaction, and separation	30 min	10^2^ - 10^7^
[13]	No	*E*. *coli*	Engineered human opsonin- MBL	No	-	Human blood	Separation	> 1 h	10^4^
[14]	No	*S*. *aureus* and *E*.* coli*	bis-Zn-DPA	No	-	Bovine diluted blood	Separation	-	10^6^
[15]	No	*E*. *coli*	Anti *E*. *coli* antibody	-	-	Buffer with Human RBCs	Separation	-	10^6^
[16]	Yes (device)	*E*. *coli*	Anti *E*. *coli* antibody	Yes	*S*.* aureus,*	Diluted Human blood	Separation	1 h	10^4^
[17]	Yes (device)	*E*. *coli*	Anti *E*. *coli* antibody	Yes	*S*.* aureus* *Salmonella* *enteritidis*	Human blood	Separation	1 h	10^5^

magnets for *E. coli* separation from blood

Legend: *bis-Zn-DPA* zinc-coordinated bis(dipicolylamine), *MBL* mannose-binding lectin, *RBP* receptor binding protein, *RBCs* red blood cells, *LOD* limit of detection

*Assay time comprises the mixing of bacteria and MNPs, reaction and separation with the permanent magnet

Despite the high efficiency of the developed device to concentrate low loads of bacteria, it is limited by the low volume that it can afford, corresponding to the recovery of 10^3^ CFU mL^−1^ of *E*. *coli* from whole blood. The system can be scaled-up to comprise higher volumes of blood, but considering the low bacterial loads reported in the BSIs (1–100 CFU mL^−1^) [32], the full recovery of bacterial cells can be challenging. A more reliable alternative would be to perform a pre-enrichment step for bacterial growth before loading the blood sample in the device to avoid the occurrence of false negatives. In this case, the time required for *E*. *coli* growth could be reduced significantly in comparison with the conventional BC since as few as 10^3^ CFU mL^−1^ would be needed for the efficient separation in the serpentine. For use as a POC, this step could be executed in a microfluidic module with an incubation chamber integrated into the serpentine device.

Overall, the total assay time using the serpentine device developed herein is a significant reduction in the blood sample preparation compared with the gold standard BC [3–5]. Also, the ability of the device to recover mostly the target bacterium can eliminate the necessity of a lengthy identification process for the invading microorganism. In BSIs, this is crucial to rapidly identify and drug-profile the pathogenic organism to introduce timely and adequate treatment. Given the fact that the survival probability for sepsis patients falls with each passing hour [7], this is a potentially life-saving solution.

Moreover, and conversely to the systems mentioned in Table 1, our device performs both the magnetic labelling of bacteria in blood and their concentration on-chip. This makes the process more rapid and standardized, enabling to integrate a miniaturized detection system to the microfluidic device for obtaining an autonomous POC. The most straightforward approach would be to employ magnetoresistive sensors that detect the fringe field of the bacteria-MNPs conjugates [30]. Another alternative is the use of fluorescent RBPs [19] to provide an optical-based detection system. So far, mainly microscopes have been used to read the fluorescence signal from labelled target antibody-MNP conjugates [33]. A step forward towards miniaturization and portability is the use of optical fibre spectrometers [33] or laser-photomultiplier systems [34]. The integration of photodiodes coupled with the MNPs would be a viable solution to complete a POC design [35].

## Conclusions

A new microfluidic system based on the use of MNPs was designed and optimized for the specific separation of *E*. *coli* from blood. Using a phage RBP as a recognition element immobilized on the surface of MNPs guaranteed the efficiency of the system for trapping the target bacterium, enabling the rapid separation of low bacterial loads from blood. This sample preparation device presents an advantage over the time-consuming BC-based methodologies for bacterial enrichment in blood. Furthermore, the recent identification of novel RBPS as probes for different bacterial species enables to easily fine-tune the system to recognize different pathogens prevalent in BSIs. Also, by using several of these microfluidic structures in parallel, targeted towards the most common sepsis-causing microorganisms, would enable obtaining a more complete picture of the generalized infection. Further coupling the presented serpentine channel with a dedicated sensing element would lead to the development of a stand-alone POC device that could significantly reduce the time and cost of diagnosis in BSIs leading to a better overall prognosis for the affected patients.

## Supplementary Information

Below is the link to the electronic supplementary material.

Supplementary file1 (DOCX 2.40 MB)

## Data Availability

The authors confirm that the data supporting the findings of this study are available within the article and its supplementary material. Raw data supporting the findings of this study are available from the corresponding author on request.
