# Supplementary material for: A magnetic nanoparticle-based microfluidic device fabricated using a 3D-printed mould for separation of Escherichia coli from blood
Source: Mikrochim Acta. 2023 Aug 18;190(9):356. doi: 10.1007/s00604-023-05924-7 (PMC10439042; doi:10.1007/s00604-023-05924-7)
Supplement: Supplementary file 1 — Supplementary file1 (DOCX 2.40 MB) [file 604_2023_5924_MOESM1_ESM.docx]

**Supplementary information**

**A magnetic nanoparticle-based microfluidic device fabricated using a 3D-printed mould for separation of *Escherichia coli* from blood**

Agnieszka Jóskowiak^1,2,3#^, Catarina L. Nogueira^3,4#^, Susana P. Costa^1,2,3,4#^, Alexandra P. Cunha^1,2,3^, Paulo P. Freitas^3,4^, Carla M. Carvalho^3*^

1. Centre of Biological Engineering, University of Minho, Campus de Gualtar, 4710-057, Braga, Portugal
2. LABBELS –Associate Laboratory, Braga and Guimarães, Portugal
3. International Iberian Nanotechnology Laboratory, Av. Mestre José Veiga s/n, 4715-330, Braga, Portugal
4. Instituto de Engenharia de Sistemas e Computadores – Microsistemas e Nanotecnologias (INESC MN) and IN – Institute of Nanoscience and Nanotechnolnology, Rua Alves Redol, 9 1000-029 Lisbon, Portugal

*Corresponding author: [carla.carvalho@inl.int](mailto:carla.carvalho@inl.int)

# These authors contributed equally to this manuscript

**
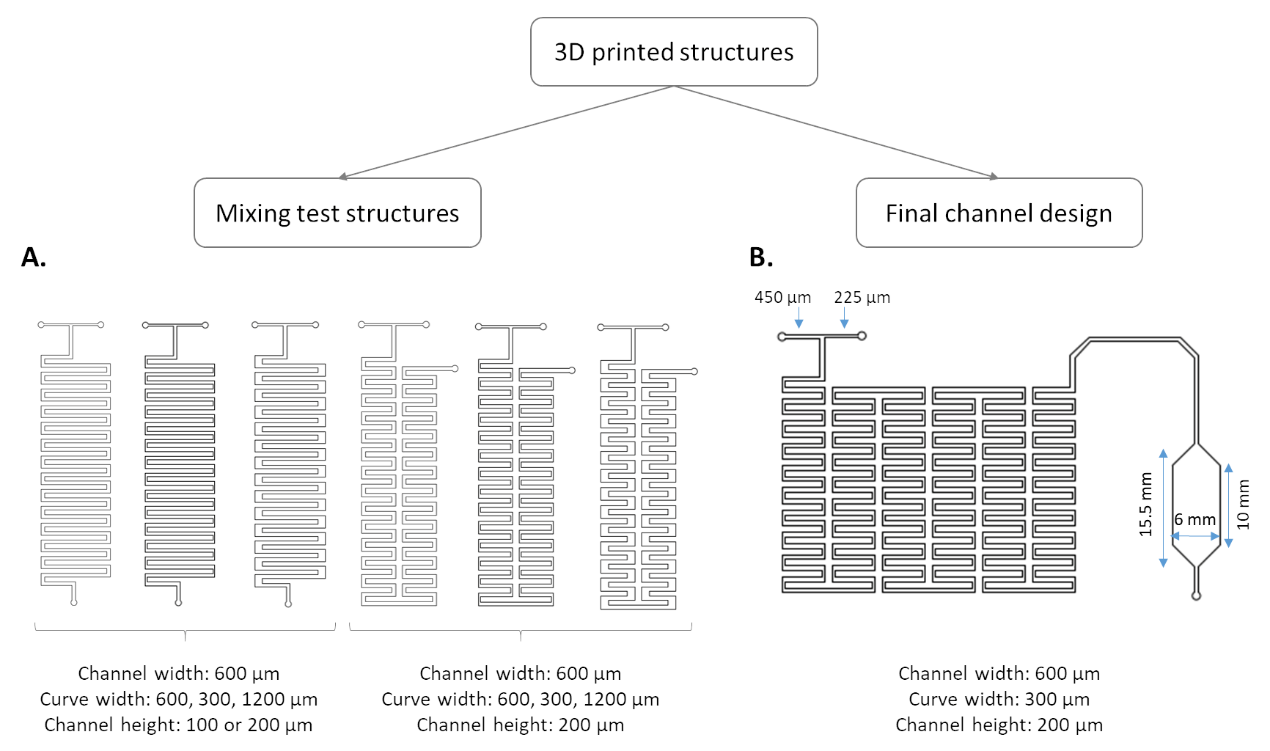
**

Figure S1. Designs of all SLA printed molds together with respective dimensions: (A.) test serpentine structures for mixing optimization assays and (B.) final design of the microfluidic mixing/trapping platform.

**
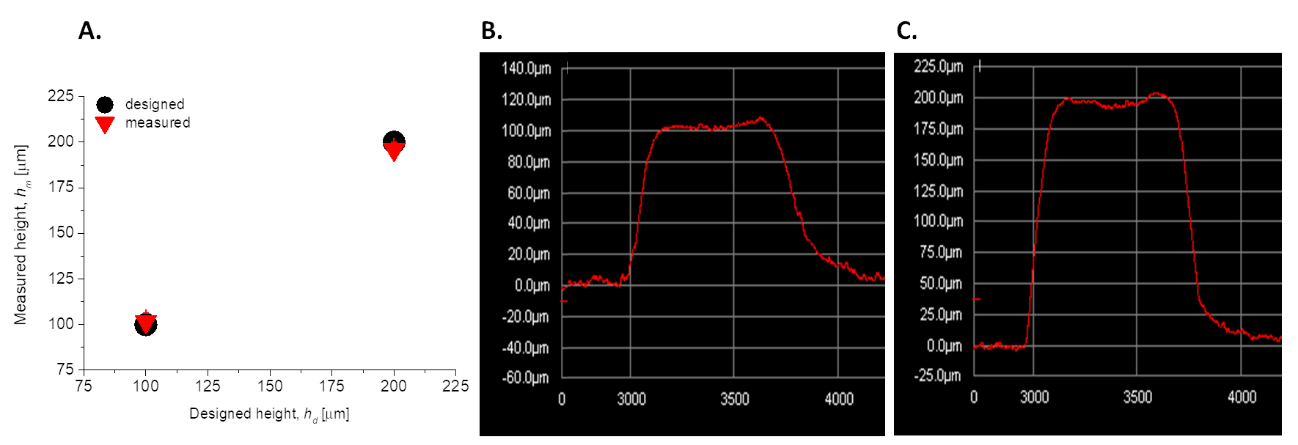
**

Figure S2. A. Measured height of the printed structures compared with the designed channel dimension. B. Profile of 100 µm high channel mold. C. Profile of 200 µm high channel mold.

**COMSOL SIMULATION AND TESTING WITH THE FOOD COLORANTS**

Three channels were designed with the curves having the same width as the rest of the channel (600 µm), half the width (300 µm), and double the width (1200 µm). All three curve types were tested in channels with two different heights: 100 µm and 200 µm, with the channel width fixed at 600 µm, to evaluate the influence of geometry on the mixing efficiency.

COMSOL simulations clearly showed that full mixing was achieved in all channel designs. However, it was far more efficient in the 200 µm high structures since it occurred earlier than in the 100 µm high channels (Figure S3). Furthermore, the reduction in the curve width to half of that of the channel (reduction from 600 µm to 300 µm) increased the chaotic liquid movement (Figure S3) and the flow velocity in that area (Figure S4) contributing to the overall mixing efficiency. This was not observed in the serpentines with curves of the same width as the channel (600 µm), where the velocity remained constant. On the other hand, for the serpentine with curves double the width of the channel (1200 µm), the velocity decreased significantly, creating dead zones, i.e. areas with low velocity and almost no flow in the corners of the curves.

Channels with an increased number of curves, with a smaller distance between them, did not show a significant change in the mixing efficiency. However, a higher number of curves per channel guarantees permanent chaotic movement throughout the whole structure and increases the probability of effective labelling of all bacteria present in the sample.

The results obtained with food colorants showed good agreement with the simulation data. The 100 µm high channel (Figure S5A) showed significantly lower mixing efficiency when compared with the 200 µm high channel (Figure S5B). For both channel heights, the reduced curve width was the one that most contributed to improving the mixing. In the case of 1200 µm curve width, the dead zones observed in the simulations manifested as an accumulation of air bubbles in the zones with the lowest flow, visible as dark spots in the corners of the channel. The same results were obtained for the serpentines with an increased number of curves (Figure S6).


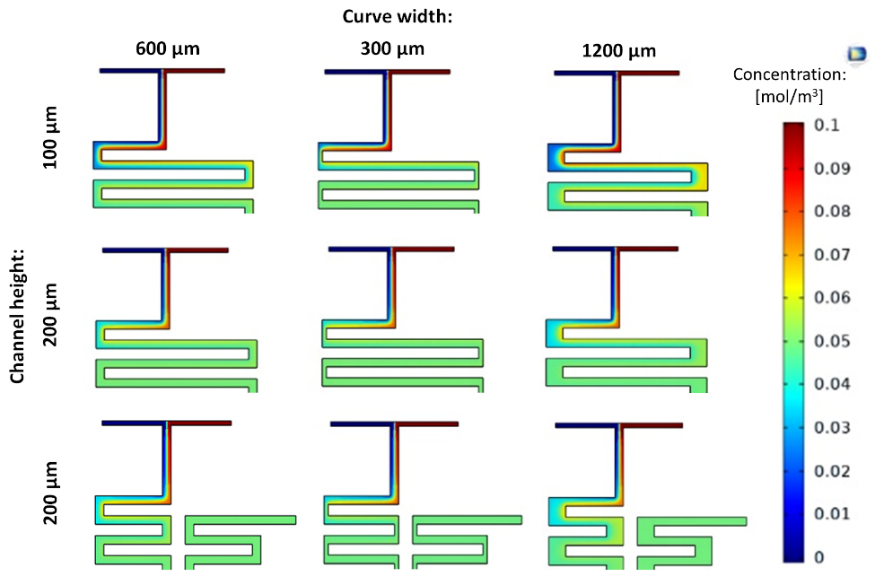


Figure S3. COMSOL mixing efficiency simulation results for inlet zone of 600 µm wide channel with different heights (100 and 200 µm), curve widths (600 µm, 300 µm, and 1200 µm), and number of curves.


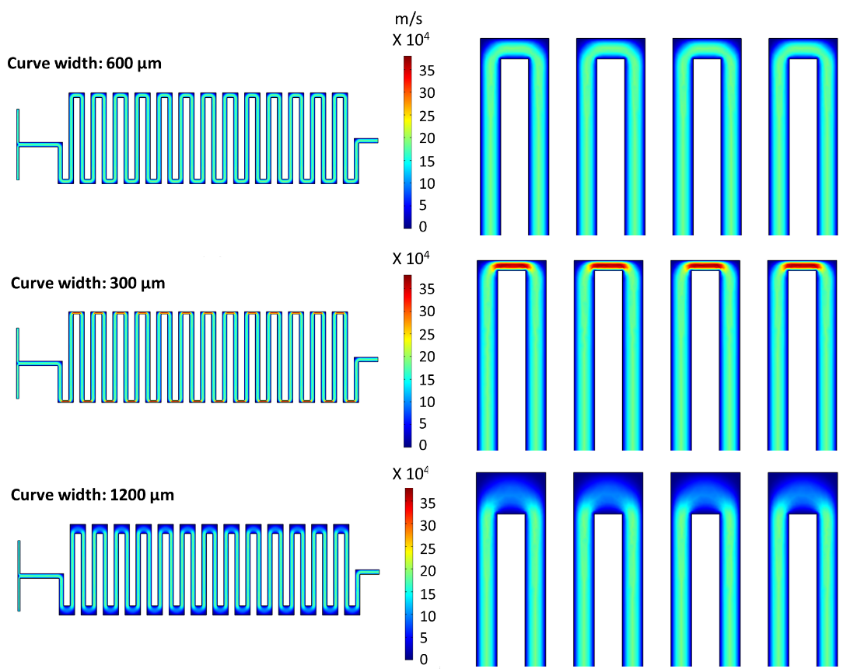


Figure S4. COMSOL simulation results of flow velocity profile in the serpentine microfluidic structures, with the channel cross-section of 600 µm by 200 µm and varying curve width: 600 µm – equal to the channel width, 300 µm – half the channel width and 1200 µm – double the channel width. Zoom on the respective channel curves shows no change in flow velocity for the 600 µm curve, the nozzle effect upon reaching the reduced channel width on the curves with 300 µm curves, and dead zones with almost zero velocity in the curves with 1200 µm.


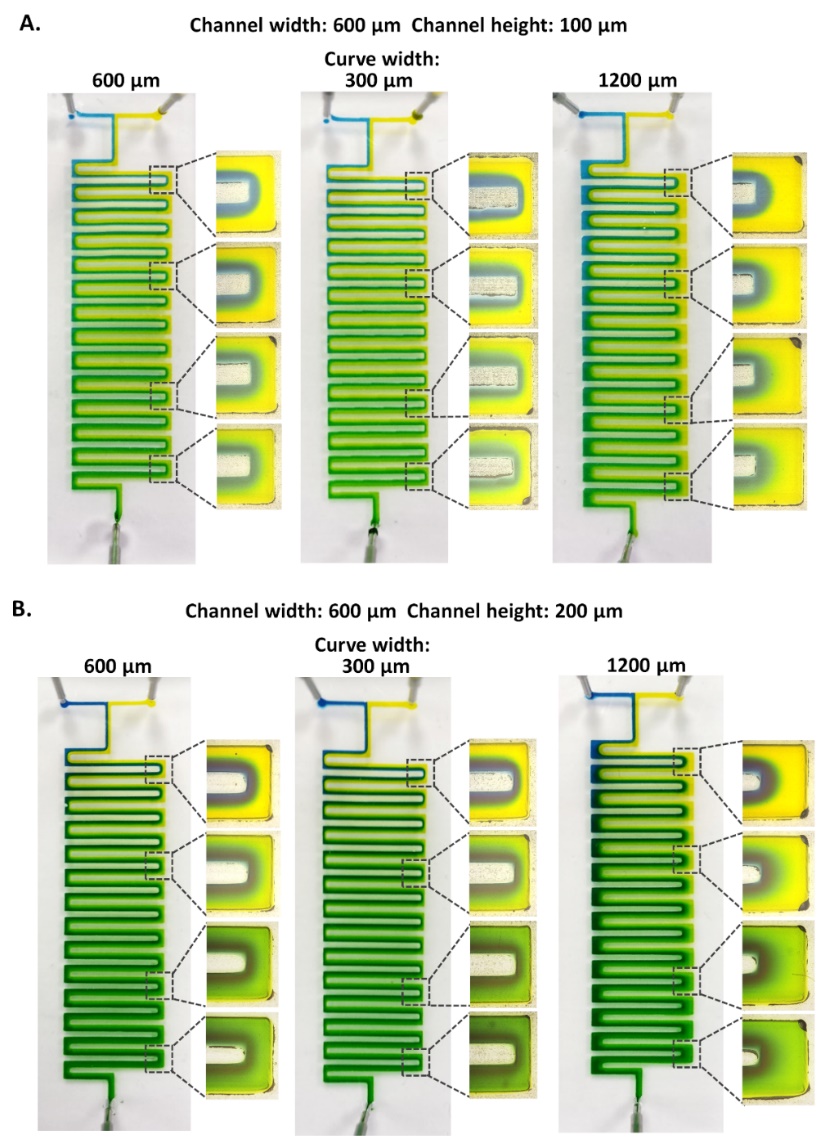


Figure S5. Differences in mixing efficiency visualized using food colorants for 600 µm wide and 100 um high (A.) and 200 µm high (B.) serpentine channels for varying curve width, from the left: 600 µm, 300 µm, and 1200 µm.

**
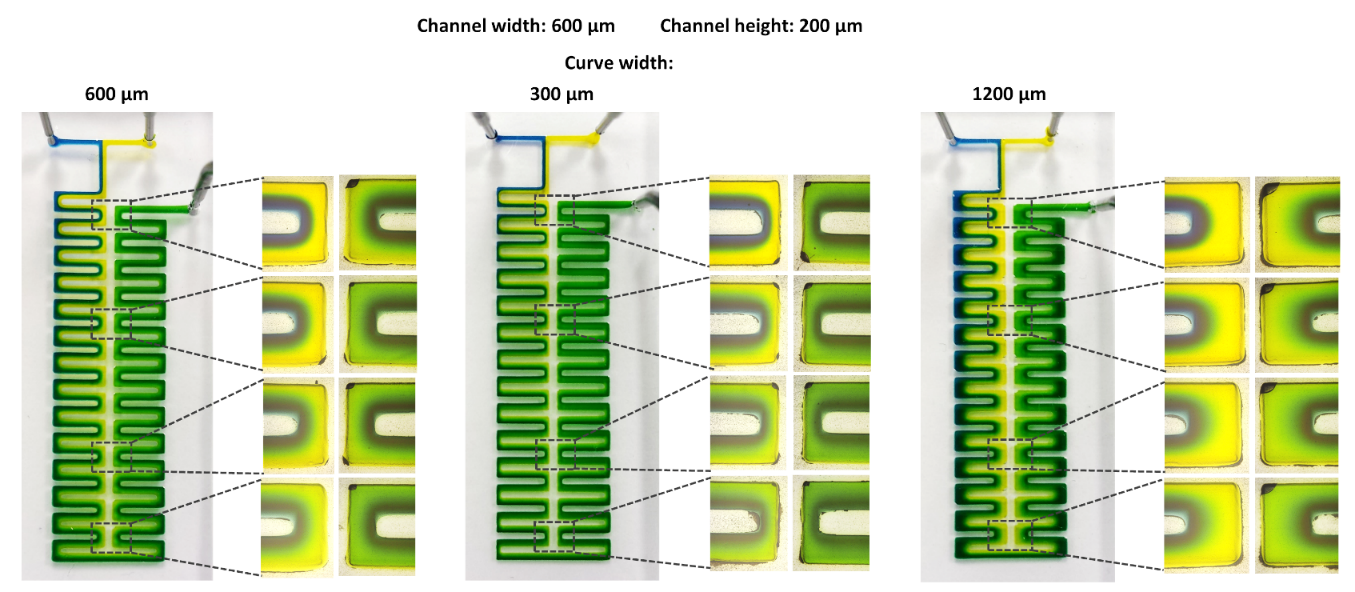
**

Figure S6. Mixing efficiency, visualized using food colorants, in 600 µm wide and 200 µm high test serpentines with an increased number of curves with varying geometry: same width as the channel (600 µm), half the width of the channel (300 µm), and double the width (1200 µm).

**

**

Figure S7. Trapping efficiency in bulk and in the microfluidic structure for assays performed with spiked 10X diluted synthetic blood for target bacteria, E. coli, at different cell concentrations and the negative control, S. aureus, at 10^7^ CFU mL^-1^.
